# Synchrony of Eukaryotic and Prokaryotic Planktonic Communities in Three Seasonally Sampled Austrian Lakes

**DOI:** 10.3389/fmicb.2018.01290

**Published:** 2018-06-15

**Authors:** Christina Bock, Michaela Salcher, Manfred Jensen, Ram Vinay Pandey, Jens Boenigk

**Affiliations:** ^1^Biodiversity, Faculty of Biology, University of Duisburg-Essen, Essen, Germany; ^2^Limnological Station, Institute of Plant and Microbial Biology, University of Zurich, Zürich, Switzerland; ^3^Institute of Hydrobiology, Biology Centre CAS, České Budějovice, Czechia; ^4^Institut für Populationsgenetik, Veterinärmedizinische Universität Wien, Vienna, Austria; ^5^Center for Hematology and Regenerative Medicine, Department of Medicine, Karolinska Institutet, Karolinska University Hospital Huddinge, Stockholm, Sweden

**Keywords:** eukaryotic plankton, prokaryotic plankton, diversity, Seasonality, freshwater, protist

## Abstract

Freshwater systems are characterized by an enormous diversity of eukaryotic protists and prokaryotic taxa. The community structures in different lakes are thereby influenced by factors such as habitat size, lake chemistry, biotic interactions, and seasonality. In our study, we used high throughput 454 sequencing to study the diversity and temporal changes of prokaryotic and eukaryotic planktonic communities in three Austrian lakes during the ice-free season. In the following year, one lake was sampled again with a reduced set of sampling dates to observe reoccurring patterns. Cluster analyses (based on SSU V9 (eukaryotic) and V4 (prokaryotic) OTU composition) grouped samples according to their origin followed by separation into seasonal clusters, indicating that each lake has a unique signature based on OTU composition. These results suggest a strong habitat-specificity of microbial communities and in particular of community patterns at the OTU level. A comparison of the prokaryotic and eukaryotic datasets via co-inertia analysis (CIA) showed a consistent clustering of prokaryotic and eukaryotic samples, probably reacting to the same environmental forces (e.g., pH, conductivity). In addition, the shifts in eukaryotic and bacterioplanktonic communities generally occurred at the same time and on the same scale. Regression analyses revealed a linear relationship between an increase in Bray–Curtis dissimilarities and elapsed time. Our study shows a pronounced coupling between bacteria and eukaryotes in seasonal samplings of the three analyzed lakes. However, our temporal resolution (biweekly sampling) and data on abiotic factors were insufficient to determine if this was caused by direct biotic interactions or by reacting to the same seasonally changing environmental forces.

## Introduction

Freshwater planktonic communities comprise an enormous diversity of different prokaryotes and protists, whereby the term “protist" refers to a non-systematic group of unicellular eukaryotes. Their phylogenetic diversity is tremendous compared to metazoans and embryophytes and they play a central role in the ecosystem (Moreira and López-Garciá, 2002; Stoeck and Epstein, 2003; Bass and Cavalier-Smith, 2004; Countway et al., 2005). In lakes and rivers, these microbes generate biomass which is used by other members of the aquatic food web. Predatory protists channel carbon and nutrients from prokaryotes and other microbes to higher trophic levels (Cho and Azam, 1990; Caron et al., 1995). Heterotrophic flagellates are main predators of bacteria and thus affects the bacterial biomass and community composition (Boenigk and Arndt, 2002; Pernthaler, 2005). On the other hand, primary producers such as algae are responsible for most of the global primary production (Falkowski et al., 1998, 2004; Field et al., 1998).

A general consensus on phytoplankton and zooplankton is that although plankton composition might differ between lakes, the overall pattern of larger taxonomic groups shows the same seasonal succession, independent of the studied lake, as long as the chemical and physical parameters are similar. Sommer et al. (1986, 2012) and Sommer (1989) introduced the PEG model, a step-by-step model for predicting phytoplankton and zooplankton seasonal occurrence in an idealized “standard” lake. Using their model, they predict that fast-growing algae such as Cryptophyceae and small centric diatoms develop at the end of winter and form a spring bloom. Grazing by herbivorous zooplankton leads to the “clear water” equilibrium, followed by a complex mixture of species. Population densities and species composition of zooplankton fluctuate throughout the summer, being also influenced by temperature. The number of diatoms increase in autumn and an autumnal maximum of zooplankton is possible due to reduced fish predation. Algal biomass then declines as winter sets in.

Numerous direct and indirect complex interactions between protists and bacteria in the microbial loop are recognized (Pomeroy, 1974; Azam et al., 1983; Pernthaler and Posch, 2009; Salcher, 2014). In lakes, top-down regulations such as selective grazing by mixotrophic and heterotrophic protists have a direct influence on community structure as well as on the abundances of different prokaryotic taxa (Pernthaler, 2005; Grossart et al., 2008; Šimek et al., 2013). On the other hand, dissolved organic substances released by phytoplankton are an important source of high quality carbon for bacteria (Cole, 1982; Hama and Handa, 1987; Cole et al., 1992; Šimek et al., 2011; Horňák et al., 2017) and have (as a substrate source) a direct effect on the prokaryotic community structure (Teeling et al., 2012; Williams et al., 2013; Shabarova et al., 2017). These top-down and bottom-up interactions result in an immense diversity and high density of prokaryotes in freshwater systems (Newton et al., 2011). While the overall diversity of prokaryotes in freshwater lakes is well known (Newton et al., 2011; Salcher, 2014), time-series data and seasonally recurrent distribution patterns have not been investigated thoroughly, and the studies have been restricted to single lakes (Salcher et al., 2008; Eiler et al., 2012; Li et al., 2015; Denef et al., 2016; Okazaki and Nakano, 2016; Woodhouse et al., 2016; Neuenschwander et al., 2018), distinct seasonal phases (Zeder et al., 2009; Eckert et al., 2012; Bižić-Ionescu et al., 2014), or to selected microbial populations (Allgaier and Grossart, 2006; Šimek et al., 2008; Salcher et al., 2011, 2015; Okazaki et al., 2013; Coci et al., 2015; Shabarova et al., 2017).

In the present study, 454 pyrosequencing of SSU rRNA amplicons was used to study the seasonal diversity of prokaryotic and protistan communities in three distinct lakes. The aim was to find out if seasonal shifts in prokaryotic and eukaryotic plankton communities occur at the same times and to the same extent. We further investigated how elapsed time correlates with changes in the similarity of plankton communities.

## Materials and Methods

### Sampling

The molecular diversity and seasonality of the planktonic organisms of lakes Fuschlsee, Wallersee, and Augstsee (all located in Salzkammergut, Austria; for more details see **Table 1**) was evaluated based on sampling in 2006 and for Lake Fuschlsee additionally in 2007 (for additional publications on this dataset, see (Medinger et al., 2010; Nolte et al., 2010; Grossmann et al., 2016). Briefly, in 2006, Lake Fuschlsee (FU) and Lake Wallersee (WA) were sampled every 2 weeks from week 16 (mid of April) until week 50 (mid of December); Lake Augstsee (AU) was sampled from weeks 28 to 44. Between March 2007 and October 2007, samples were taken from Lake Fuschlsee every 3 weeks (ten samples in total, starting in week 13 at the end of March). For the whole sampling period, integrated water samples covering the upper 10 m of the water column (epilimnion) within the pelagic zone were collected. Samples were filtered onto 0.2 μm polycarbonate filters, air dried and then frozen at -80°C until further processing. Details on dates and environmental data are summarized in Supplementary Table S1. The samples in following chapters of the manuscript are labeled according to the lake of origin and the sampling week. In the case of sampling of Lake Fuschlsee in 2007, the number 07 is attached to the week (e.g., FU1307 for Lake Fuschlsee week 13 in the year 2007).

**Table 1:** Details on the sampled lakes.

	Fuschlsee (FU)	Wallersee (WA)	Augstsee (AU)
Location	47°48′10″N, 13°16′20″E	47°39′49.27″N, 13°47′10.43″E	47°39′49″N, 13°47′10″E
Altitude (m a.s.l.)	663	506	1643
Max. depth (m)	67.3	23	8.2
Volume (10^6^ m^3^)	97.9	76.6	n.a.
Trophic state	oligo-mesotrophic	meso-eutrophic	n.a.
Eukaryotic OTUs (2006/2007 in FU)	1383 (871/1127)	964	716
Prokaryotic OTUs (2006/2007 in FU)	2529 (2207/1291)	1987	727

### Sample Preparation for Next Generation Sequencing

Sample preparation and next generation sequencing strategies are described in detail in Grossmann et al. (2016). In brief, genomic DNA was extracted from the 0.2 μm polycarbonate filters with the DNeasy Tissue kit (Qiagen Gmbh Hilden, Germany) and amplified using HPLC purified PCR primers. For the V9 region of the eukaryotic SSU rRNA gene, the forward primer 1391f (5′-GTACACACCGCCCGTC-3′) (Stoeck et al., 2010) and reverse primer Euk B (5′-TGATCCTTCTGCAGGTTCACCTAC-3′) (Medlin et al., 1988) were used, following the protocol of Nolte et al. (2010). For the prokaryotic dataset, primer 1492-rm (5′-GNTACCTTGTTACGACTT-3′; (Baker et al., 2003; Roesch et al., 2007) and forward primer 5′-GGTTAAGTCCSGYAACGA-3′ (Greisen et al., 1994; Dupont, 2016) were used. PCR products were pooled and sequenced on a 454 Roche FLX sequencer. The eukaryotic reads are published at the NCBI database under the Bioproject Accession number PRJNA384347^1^; the prokaryotic reads are published under the Bioproject Accession number (PRJNA445789).

### Bioinformatic Analyses

The resulting sequences were quality filtered including adapter and primer clipping, removal of sequences with ambiguous bases (Ns), a quality score <24 when averaged across the read after clipping adapters and primers or a minimum sequence length below 200 bp (including PCR primers) (Nolte et al., 2010; Pandey et al., 2010). Chimera check was done by using UCHIME (Edgar et al., 2011). Sequences which passed the quality filtering were clustered in OTUs by using Swarm (version 2.1.6) with default settings (Mahé et al., 2014) and singletons were removed. Taxonomic assignment for the eukaryotic dataset was done by using BLASTn (version 2.3.0) against the NCBI nucleotide database. For each blast hit, the 10 first results were compared and the one with the most detailed taxonomic assignment was used (pident minimum 85.0, evalue 1e12). No detailed taxonomic resolutions were used for any downstream analyses (focusing on higher taxonomic levels only). OTUs of Metazoa and Embryophyta were excluded. The resulting dataset consisted of 1,851 eukaryotic OTUs with a total of 216,349 reads (53 samples in total). Prokaryotic OTUs were blasted against the SILVA database (SILVA release 123 July 23, 2015). This resulted in 3,624 OTUs with a total of 70,836 reads (samples with fewer than 100 reads were excluded). Prokaryotic OTUs were further manually assigned to ubiquitous freshwater lineages as proposed by Newton et al. (2011) or summed up on the genus level for described taxa.

### Chemical and Environmental Parameters

Lake water temperature, conductivity and pH value were determined for each sampling date (Supplementary Table S1). The additional factors alkalinity (alkal), nitrate (NO_3_), sulfate (SO_4_), chloride (Cl^-^), ammonium (NH_4_), sodium (Na), potassium (K), magnesium (Mg), calcium (Ca), dissolved reactive silica (DRSi), total phosphorous (TP), dissolved organic carbon (DOC), dissolved nitrogen (DN), and dissolved phosphorous (DP) were only measured once for each lake as seasonal fluctuations were considered to be small and largely within the measurement errors (Landesregierung, 2009). Details on measured values can be found in Supplementary Table S2.

### Diversity and Multivariate Statistical Analyses

Standardization among samples was performed by randomly subsampling the table of OTUs to the minimum read level (247 for eukaryotic samples, 109 for prokaryotic samples) using the rrarefy function of the R package vegan (Oksanen et al., 2011). This procedure was repeated 100 times for the eukaryotes and 1000× for the prokaryotic dataset. The resulting dataset was used for all further downstream analyses. Multivariate statistics and diversity estimates were performed using the R vegan package (Oksanen et al., 2011). Abundance tables were subjected to Hellinger transformation prior to Bray–Curtis dissimilarity matrices for the respective analyses. To identify patterns in community composition, a principle coordinates analysis (PCoA) was carried out and the function *envfit* of the package vegan and gclus in R studio (Oksanen, 2015) was applied with 999 permutations to check for the correlation between its main axes and the factors temperature, conductivity, pH, and sampling week. The *envfit* method does not explain or contribute to the (PCoA) ordination. However, it is suitable to look for a possible significant integration of environmental factors in an ordination. Hierarchical clustering of Bray–Curtis dissimilarity was performed by using ward.d2 clustering and hcoplot.R source with the optimal number of clusters (3) determined by the Rousseeuw quality index (Borcard et al., 2011). We used the co-inertia analysis (CIA) to recognize a potential synchrony of the prokaryotic and eukaryotic datasets. CIA can identify co-relationships between datasets with the same samples via multivariate analyses. The results are graphically displayed in a bi-plot; the projections are connected by an arrow whereby the length of the line indicates the divergence between the two datasets. CIA were conducted in R with the ade4 version 1.6-2 package (Dray and Dufour, 2007; Thioulouse and Dray, 2007) based on PCoA analyses. Linear correlations between elapsed time between samples (differences in weeks) and Bray–Curtis dissimilarities were determined by linear regression on Bray–Curtis dissimilarities of community composition (dependent variable) versus square root of time differences in weeks.

## Results

### Taxonomic Composition of the Eukaryotic Samples

**FIGURE 1:**
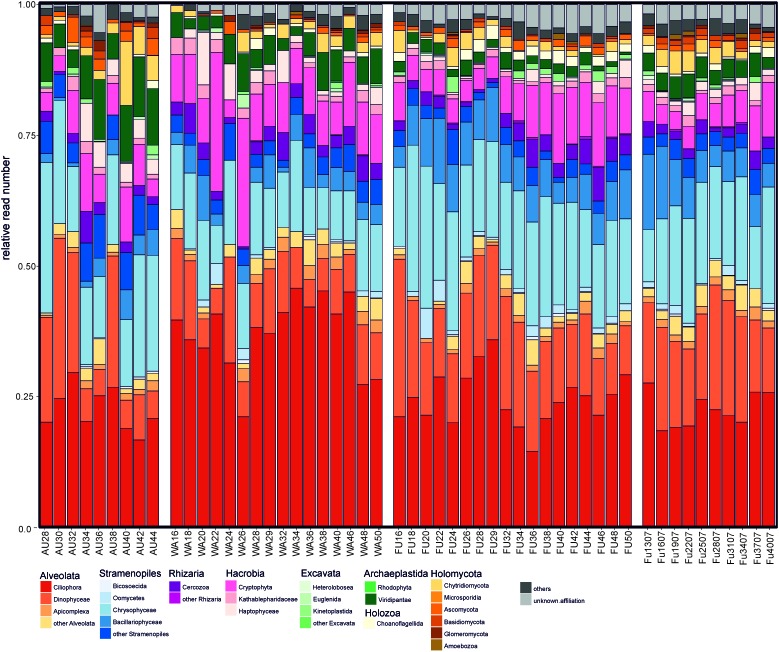
Taxonomic composition of the analyzed eukaryotic samples based on Hellinger transformed rarefied reads.

On an annual average, samples from Lake Augstsee consisted of 40% of Alveolata reads, of which the majority were affiliated with Ciliophora (22.6% of the reads) and Dinophyceae (14.4% of the reads), and only to a lesser extend (<2% of reads) to Apicomplexa and Perkinsea (**Figure 1**). An average of 28% of the reads belonged to Stramenopiles, with Chrysophyceae as the dominating group (18.6% of the reads). Bacillariophyceae (3% of the reads) and Eustigmatophyceae played only minor roles. Cryptophyta gained only 7% of annual average reads with Cryptomonadales as the main lineage. All other groups occurred to a lesser extent (<5% of reads). A high amount of Alveolata reads could be found in Lake Wallersee (51% of the reads), again with Ciliophora (37% of the reads) and Dinophyceae (10% of the reads) as main lineages. Further dominant groups were Stramenopiles with 20% of encountered reads, dominated again by Chrysophyceae (12.7% of the reads) and Bacillariophyceae (4% of the reads) and Cryptophyta with 11% of obtained reads. Lake Fuschlsee showed a similar composition during the 2 years. Alveolata dominated with 44% of the reads (Ciliophora 24.6% of the reads; Dinophyceae 15.9% of the reads) in 2006 and 45% of average annual reads in 2007 (Ciliophora 22.5%; Dinophyceae 17.5%), followed by Stramenopiles with 31% of the reads (19.4% Chrysophyceae, 7.5% Bacilariophyceae) and 29% of the reads (17.2% Chrysophyceae, 7.7% Bacilariophyceae). Another main group was Cryptophyta with 8% (2006) and 6% (2007) of obtained reads. The annual average reads of Viridiplantae was relative low (below 5% annual average reads) for all lakes except for Lake Augstsee, where they accounted periodically for more than 10% of the reads (annual average 8%). Within the Viridiplantae of Lake Augstsee, Chlorophyceae was the dominating group with several lineages, whereas Chlamydomonadales reached an annual average within the Viridiplantae of 33% and Sphaeropleales of 37%. A small number of reads could not be assigned to any group, i.e., 1% of reads in lakes Augstsee and Wallersee, and 2 and 3% in Lake Fuschlsee in 2006 and 2007. A barchart showing the relative abundances of the respective groups during the year are shown in **Figure 1** and Supplementary Figures S1A–D.

### Taxonomic Composition of the Prokaryotic Samples

**FIGURE 2:**
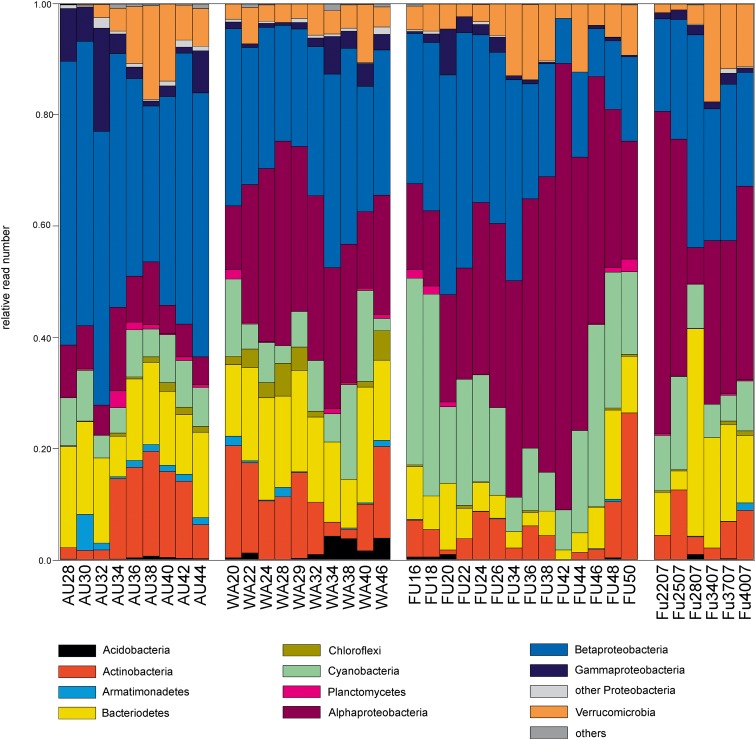
Taxonomic composition of the analyzed prokaryotic samples based on Hellinger transformed rarefied reads.

The most abundant prokaryotes in all three lakes were affiliated with the phyla Proteobacteria, Actinobacteria, Bacteroidetes, Verrucomicrobia, and Cyanobacteria, and to a lesser extent with Acidobacteria, Armatimonadetes, Chloroflexi, and Planctomycetes (<2% of reads each), however, in a lake-specific fashion (**Figure 2**). Lake Augstsee was dominated by Betaproteobacteria (44% of obtained reads), followed by Bacteroidetes (14% of the reads), and Actinobacteria (10% of the reads), while all other phyla contributed <10% of reads in an annual average. In contrast, samples from lakes Wallersee and Fuschlsee contained less Betaproteobacteria (27 and 24% of the reads, respectively) and more Alphaproteobacteria (25 and 35% of the reads, respectively). The majority of reads from all samples could be classified to well-known ubiquitous freshwater lineages that dominated the assemblages (**Figure 2** and Supplementary Figure S2A–D). Almost all Actinobacteria were affiliated with either lineage acI (‘*Ca*. Nanopelagicales’, 4–6%, annual read average) or acIV (Acidimicrobiales, 2–5% of the reads) with conspicuous seasonal maxima of 10–14% (acI) and 7–11% (acIV) of the reads in the three lakes. Bacteroidetes were mainly represented by one dominant taxon per order, i.e., *Pseudoarcicella* (Cytophagales), *Fluviicola* (Flavobacteriales), and uncultivated lineage NS11-12 (Sphingobacteriales), which accounted for 1–4% of the reads on an annual average. Lake Fuschlsee contained high proportions of Cyanobacteria, especially *Planktothrix* sp. in spring and autumn (max: 30% of the reads) and *Synechococcus* sp. in summer (max: 19% of the reads), whereas the other two lakes did not show pronounced cyanobacterial blooms. Proteobacteria and Verrucomicrobia also displayed distinct lake-specific distribution patterns at a higher taxonomic resolution. While the ubiquitous LD12 lineage of Alphaproteobacteria (‘*Ca*. Pelagibacterales’) was highly abundant in lakes Wallersee (annual read average: 21%, max: 30% of all reads) and Fuschlsee (annual read average: 31%, max: 79%), these microbes were virtually absent in Lake Augstsee (max: 2%). Caulobacterales, Rhodobacterales, and Sphingobacteriales accounted only for minor portions of Alphaproteobacteria (annual average: 3–6% of reads). Betaproteobacteria, on the other hand, were very diverse with different members of the families Alcaligenaceae, Burkholderiaceae, Comamonadaceae, Oxalobacteriaceae, and Methylophilaceae displaying lake-specific and season-specific distribution patterns. Lake Augstsee differed from the other two lakes by having higher relative read numbers of *Polynucleobacter* sp. (annual read average: 17%, max: 27%), the freshwater lineage GKS98 (Alcaligenaceae; average reads: 2%, max: 7%), several taxa affiliated with Comamonadaceae (average reads: 15%, max: 35%), and Methylophilaceae (average reads: 7%, max: 26%). A strong lake-specificity was also observed for Verrucomicrobia, where the uncultivated vadinHA64 lineage (Opituae) was highly abundant in Lake Augstsee (average reads: 5%, max: 16%), whereas *Methylacidiphilum* sp. (Methylacidiphilales) dominated in Lake Fuschlsee (average reads: 5%, max: 14%).

### Community Structure of the Eukaryotic Data Set

**FIGURE 3:**
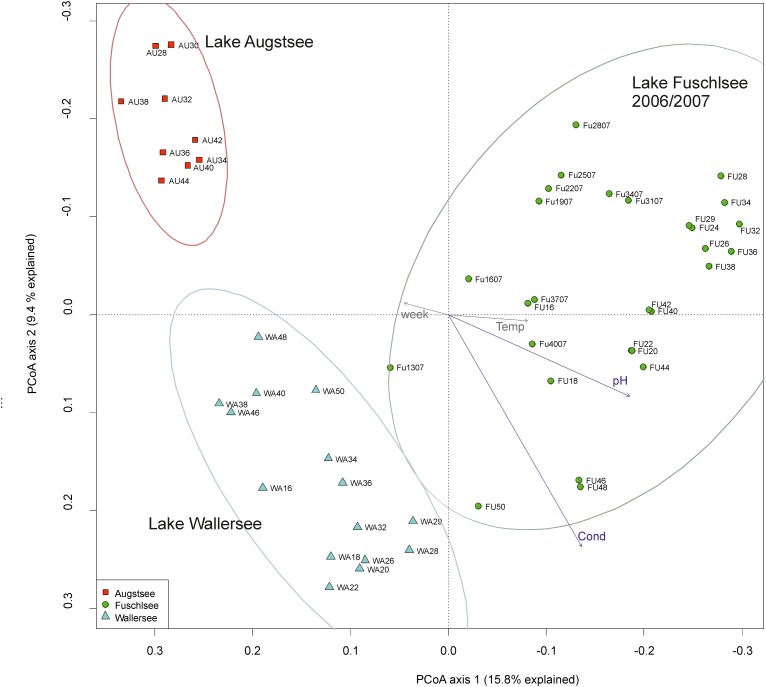
Community structure of the eukaryotic dataset by principle coordinate analyses (PCoA). The arrows of the environmental factors were calculated passively after PCoA by envfit. Factors pH and conductivity, but not temperature and week could be significantly (*p* < 0.001) integrated in the ordination of the sites.

The PCoA ordination of the eukaryotic dataset reflected a separation according to lake origin (**Figure 3** and Supplementary Figure S3). Three main clusters based on OTU composition could be observed: samples originating from Lake Fuschlsee (years 2006 and 2007), from Lake Wallersee and from Lake Augstsee. Sample Wallersee week 24 (Wa24) clustered within samples of Augstsee (Supplementary Figure S1) and was excluded from further analyses, as a possible mix-up of samples or mistakes at PCR or sequencing cannot be ruled out. This sample also had the lowest number of reads of the eukaryotic dataset. The à posterior applied environmental factors onto the PCoA revealed a positive correlation for Lake Fuschlsee with conductivity and pH. Not significant were the factors week and temperature (for details see **Figure 3**). Influence of additional environmental parameters which were measured only once during the campaign are displayed in Supplementary Figure S3A.

**FIGURE 4:**
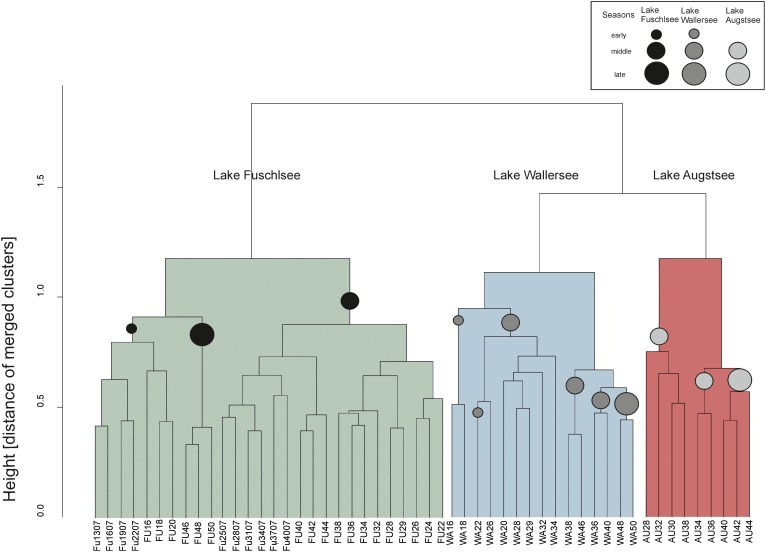
Unconstrained dendogram of the hierarchical clustering of the Bray–Curtis dissimilarity matrices of the eukaryotic dataset. The samples on the *x*-axis are labeled according to the lake of origin (WA, Wallersee, AU, Augstsee, FU, Fuschlsee) and sampling week. At the *y*-axis, the height corresponds to the distance of merged clusters based on the variance criterion of the WARD.D2 cluster method. Circles in the dendrogram indicate samples belonging to the same “season.”

**FIGURE 5:**
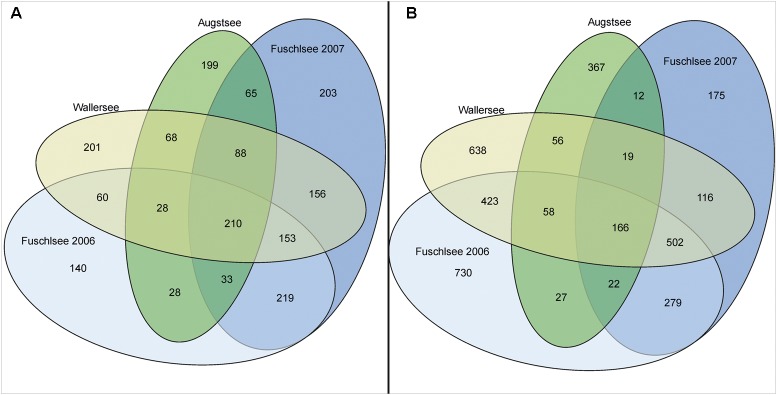
Diagram showing the overlap of occurring OTUs between the analyzed lakes. **(A)** Eukaryotic samples; **(B)** Prokaryotic samples.

The hierarchical clustering of the Bray–Curtis dissimilarity matrices further illustrated the separation and seasonality of the lakes (**Figure 4**). The three clusters already observed in the PCoA were further divided in subclusters roughly according to season. The Fuschlsee spring samples (weeks 13–22 2007, 16–20 2006) clustered next to autumn samples of 2006 (Fu46-50). The next subcluster consisting of summer samples from 2006 (weeks 22–38) was closely related to samples from October 2006 (weeks 40–44) and the remaining samples from 2007 (weeks 25–40). Samples within the Wallersee cluster grouped according to season with a cluster containing spring samples (weeks 16–32) and a cluster containing summer samples (weeks 34–50). A similar clustering (but not as strict as to weeks) was also observed for Augstsee. The Venn diagrams in **Figure 5A** and Supplementary Figure S4 show the overlap between lakes and the obtained seasonal clusters according to hierarchical clustering based on Bray–Curtis dissimilarities. Only 210 OTUs out of 1851 were shared between all lakes (11.43%). A high number of recurring OTUs could be detected in the 2-year-investigation in Lake Fuschlsee (615 shared OTUs). Details about overlapping OTUs between the different seasonal clusters can be found in Supplementary Figures S4A–D.

### Community Structure of the Prokaryotic Data Set

**FIGURE 6:**
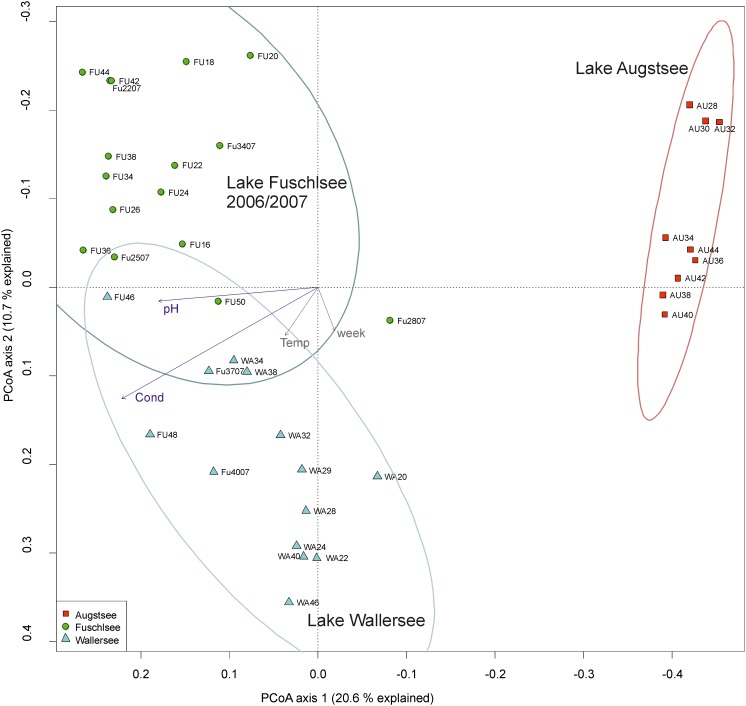
Community structure of the prokaryotic dataset by principle coordinate analyses (PCoA). The arrows of the environmental factors were calculated passively after PCoA by envfit. Factors pH and conductivity, but not temperature and week could be significantly (*p* < 0.001) integrated in the ordination of the sites.

**FIGURE 7:**
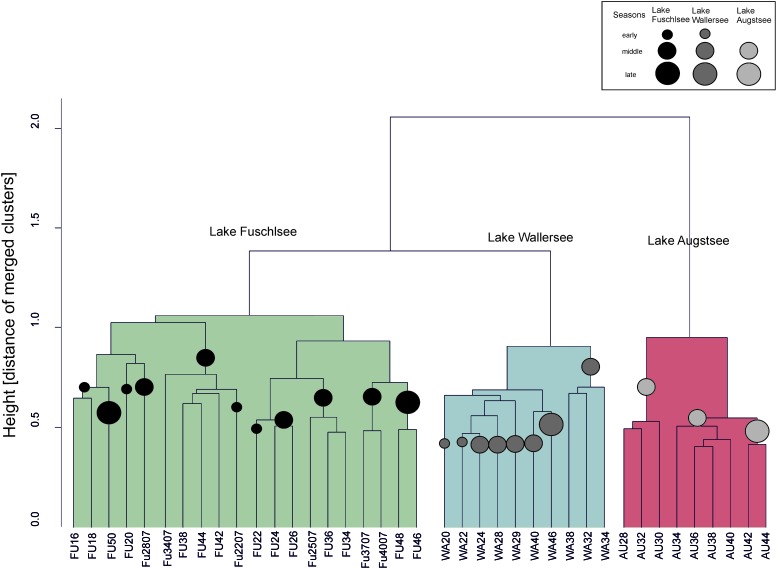
Unconstrained dendogram of the hierarchical clustering of the Bray–Curtis dissimilarity matrices of the prokaryotic dataset. The samples on the *x*-axis are labeled according to the lake of origin (WA, Wallersee, AU, Augstsee, FU, Fuschlsee) and sampling week. At the *y*-axis the height corresponds to the distance of merged clusters based on the variance criterion of the WARD.D2 cluster method. Circles in the dendrogram indicate samples belonging to the same “season.”

The PCoA of the prokaryotic dataset largely resembled the eukaryotic orientation with lake-specific clusters being significantly related to the same environmental factors, i.e., samples from Lake Fuschlsee were correlated to conductivity and pH (**Figure 6**). The influence of other environmental parameters which were measured only once during the campaign can be found in Supplementary Figure S3B. The hierarchical clustering for Lake Augstsee revealed a separation into spring samples (weeks 28–32) and the remaining samples (**Figure 7**). Samples from Lake Wallersee could be divided into a subcluster containing late-summer samples (weeks 32–38) and one large subcluster containing the remaining samples with a slight separation of the autumnal samples (weeks 40–46). Most samples from Lake Fuschlsee could not be separated according to seasons via hierarchical clustering as the years and seasons were intermixed, with the exception of one large subcluster containing summer samples from 2006 (weeks 22–36) and one sample from 2007 (Fu2507; see **Figure 7**). Each lake showed a distinct OTU composition with few overlapping OTUs between two or more lakes and only 166 OTUs out of 3590 (4.62%) were detected in all lakes (**Figure 5B**). As for eukaryotes, a large overlap was observed for samples gained from two consecutive years from Lake Fuschlsee (969 OTUs). Details on seasonally occurring OTUs can be found in Supplementary Figures S5A–C.

### Comparison of Prokaryotic and Eukaryotic Datasets

**FIGURE 8:**
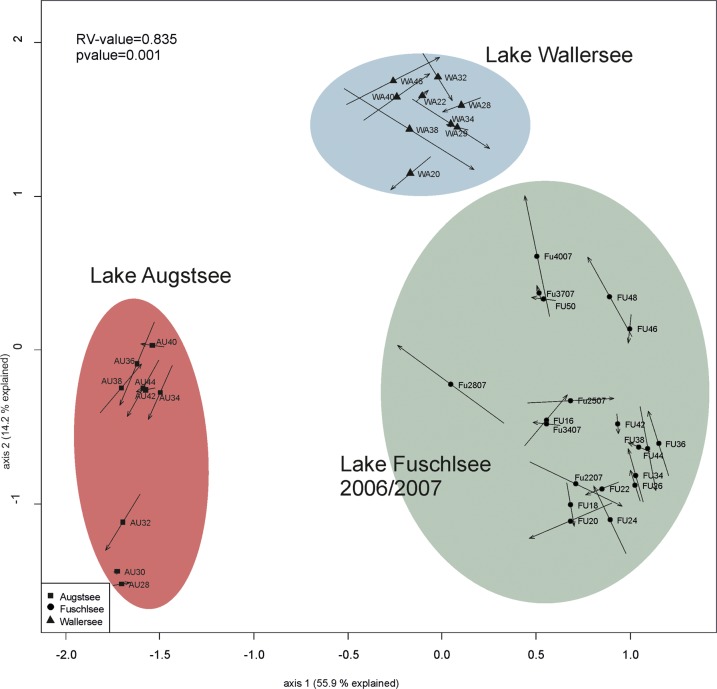
Comparison of the community structure between eukaryotic and prokaryotic dataset via Co-inertia analysis (CIA). At the beginning of the arrow is the theoretical position of the eukaryotic sample, at the end of the arrow is the position of the prokaryotic sample. Line length of the arrow indicates the divergence between the two datasets.

The comparison of prokaryotic and eukaryotic OTUs showed a very high similarity between the two datasets in the CIA with a RV value of 0.835 (**Figure 8**). The short arrows are further evidence that the prokaryotic and eukaryotic data show a highly similar pattern in terms of lake specificity and community composition over the season.

### Temporal Correlation Between Elapsed Time and Community Composition

**FIGURE 9:**
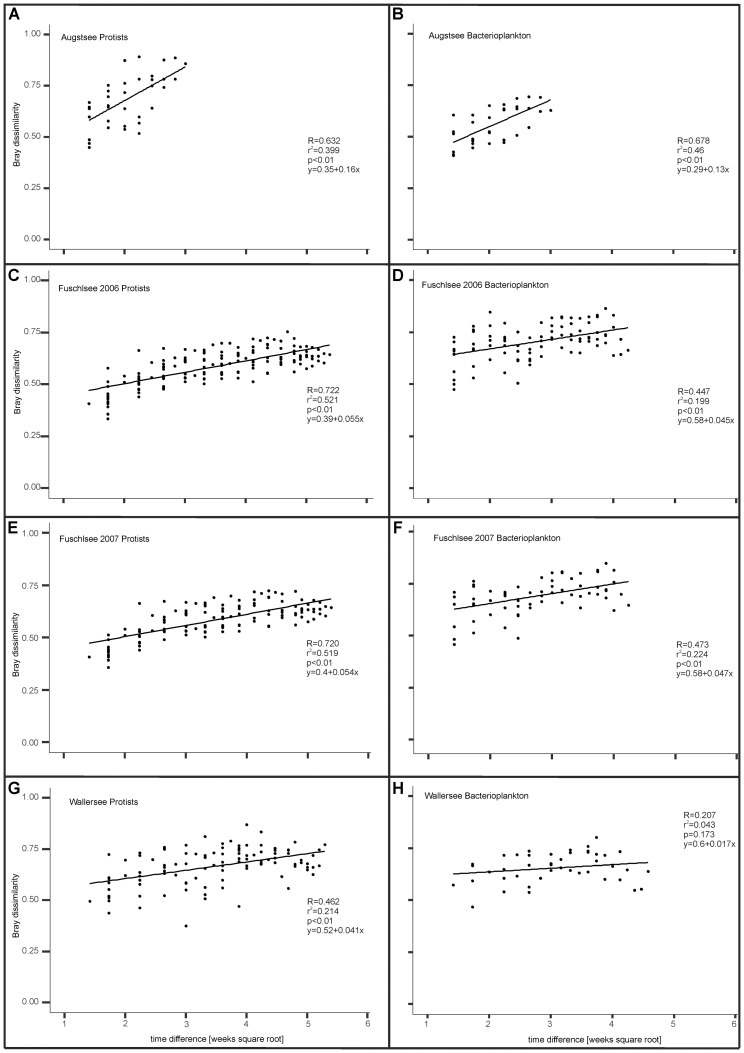
Temporal influence on Bray dissimilarity between samples (see section “Materials and Methods”). **(A)** Eukaryotic samples Lake Augstsee; **(B)**: Prokaryotic samples Lake Augstsee; **(C)**: Eukaryotic samples Lake Fuschlsee 2006; **(D)**: Prokaryotic samples Lake Fuschlsee 2006; **(E)**: Eukaryotic samples Lake Fuschlsee 2007; **(F)**: Prokaryotic samples Lake Fuschlsee 2007; **(G)**: Eukaryotic samples Lake Wallersee; **(H)**: Prokaryotic samples Lake Wallersee.

By analyzing the Bray–Curtis dissimilarities between samples within a lake, a positive linear correlation was observed between time differences between samples and corresponding Bray–Curtis dissimilarities (**Figure 9**). This indicates a directional change of the microbial communities in all lakes. The Pearson correlation and slope of the linear regression differed between the lakes and between prokaryotic and eukaryotic communities with eukaryotes generally showing a higher correlation than prokaryotes. An exception was found for Lake Augstsee, where we detected a similarly high correlation for prokaryotic and eukaryotes (*R* = 0.632 and *R* = 0.678, respectively) and slope of the linear regression (0.16 and 0.13, respectively), indicating a similar rate of change (**Figures 9A,B**). However, different values were obtained for Lake Fuschlsee; these showed a highly recurrent pattern for both years. The correlations for eukaryotes in 2006 and 2007 were similarly high (*R* = 0.722 and *R* = 0.720 respectively) with similar slopes (0.055 and 0.054) (**Figures 9C,E**), while prokaryotes showed lower linear correlations (*R* = 0.447 and *R* = 0.473) and slopes (0.045 vs. 0.047) in 2006 and 2007 (**Figures 9D,F**). Lower correlation values were observed for Lake Wallersee (**Figures 9G,H**). The correlation value for eukaryotes was *R* = 0.46 if sample week 24 (clustering between Lake Augstsee samples) was excluded and the bacterial correlation was only *R* = 0.207. As a general statement linear correlations between time difference and increase in dissimilarity were found for eukaryotes and to a lesser extent for bacteria, with varying strengths for the individual lakes.

## Discussion

In this study, high-throughput sequencing of SSU rRNA genes was used to characterize changes in eukaryotic protists and bacterioplankton communities over an ice-free period in three Austrian lakes. The 18S rDNA was chosen as a target gene for the eukaryotic protists as it contains both highly conserved regions needed for primer annealing and variable regions allowing for detailed taxonomic classification. Furthermore it amplifies easily due to high DNA copy numbers (Pawlowski et al., 2012; Guillou et al., 2013). The proportions of reads are assumed to correlate with the relative abundance of the organisms and the copy number of the marker gene (Amend et al., 2010; Medinger et al., 2010). Different gene copy numbers in particular of eukaryotic taxa affect the relative read abundance of organism groups even though gene copy number seems roughly to correlate with cell size and thus biovolume. Nevertheless, some taxa such as ciliates or Katablepharids are systematically overrepresented in molecular surveys (Zhu et al., 2005; Grujcic et al., 2018). Comparative studies on cell abundances and read abundances demonstrated, however, that abundance shifts are well resolved by read abundance data despite deviations between cell and read abundances (Medinger et al., 2010). Prokaryotic copy numbers of 16S rRNA genes vary not as drastically as 18S rRNA copies in eukaryotes (Lee et al., 2009). Small, genome-streamlined microbes like ‘*Ca*. Nanopelagicales’, LD12 Alphaproteobacteria, ‘*Ca*. Methylopumilus’, *Rhodoluna* sp., or *Polynucleobacter* sp. (Hahn et al., 2014, 2016; Salcher et al., 2015; Eiler et al., 2016; Neuenschwander et al., 2018) and most Verrucomicrobia contain only one copy per cell and might be thus slightly underrepresented in our study. On the other hand, genome-sequenced members of Cytophagales, Flavobacteriales, and Sphingobacteriales (Bacteroidetes) contain on average 3–4 copies; Caulobacteriales, Rhodobacteriales, and Sphingomonadales (Alphaproteobacteria) 2–3 copies; and betaproteobacterial families Comamonadaceae, Oxalobacteraceae, and Alcaligenaceae contain three copies of the 16S rRNA per cell (Lee et al., 2009). These microbes might be overrepresented in our dataset, however, they are also known to be larger and thus, the read number might well correspond to the biomass of these organisms (Šimek et al., 2014).

### Distinct Lake-Specific Microbial Communities

Microbial communities and their diversity are influenced by an interplay of biotic interactions and abiotic environmental conditions (Yannarell and Triplett, 2005; Martiny et al., 2006; Lindstrom and Langenheder, 2012; Boenigk et al., 2014). In particular seasonality (Bock et al., 2014; Tammert et al., 2015) and, at least for eukaryotes, also biogeography (Boenigk et al., 2018), have a strong influence on microbial communities and shape their diversity. Fluctuations in local parameters and inter-annual variations were reported to occasionally mask the seasonal changes in microbial communities (Tammert et al., 2015). Whether chemical factors, biotic interactions or a mixture of both is responsible for the clustering of the samples, however, cannot be resolved with our datasets. Freshwater microbes can have short turnover rates in the range of a few days (Zeder et al., 2009; Eckert et al., 2012; Šimek et al., 2014), thus, our fortnightly sampling scheme was not sufficient to identify short-termed biotic feedbacks. However, in both of our datasets a clear distinction between the lakes was observed, with an overlap in co-occurring OTUs between lakes of approx. 11% (eukaryotic) and approx. 5% (prokaryotic) of OTUs occurring in all lakes, respectively (**Figure 5**). The conducted CIA between the two datasets revealed a high RV value, demonstrating a strong correlation between eukaryotic protists and bacterioplanktonic communities in the different lakes (**Figure 8**). These results are consistent with the conducted PCoAs, which revealed significant correlations for both datasets with conductivity, pH, DOC, ammonium, total nitrate, and total phosphate (Supplementary Figure S3). Several of these aforementioned components are related to the trophic state index and are used for classification of different lake types (Wu et al., 2009). It is well known that lake trophic state and the concentration of nutrients has differential effects on microbial species (Llirós et al., 2014). Phosphorous and/or nitrogen are often factors limiting prokaryotic and phytoplanktonic growth (Schindler, 1977; Elser et al., 1990; Carlsson and Caron, 2001; Andersson et al., 2006; Abell et al., 2010; Carlsson et al., 2012). Not only growth rates and productivity are influenced by nutrients; nutrient enrichments can also have a tremendous effect on the composition of bacteria communities (Fisher et al., 2000; Nelson and Carlson, 2011; Peura et al., 2012). The same applies to the source of nutrients. Experiments have shown that the type of nitrogen or phosphorus source (inorganic and/or organic) strongly affects the planktonic microbial community composition and functioning (Teira et al., 2010; Rofner et al., 2016).

Biogeographic studies have revealed that high mountain lakes have a distinct community, may act as biogeographic islands and that richness and phylogenetic diversity decreases with elevation in some cases (Bryant et al., 2008; Sommaruga and Casamayor, 2009; Xing et al., 2009; Filker et al., 2016; Hayden and Beman, 2016; Boenigk et al., 2018). This is consistent with our dataset, as the high mountain lake Augstsee [1643 m above sea level (a.s.l.)] had the lowest number of prokaryotic and eukaryotic OTUs (727 and 716, respectively; **Table 1**) The most striking difference in our bacterial dataset was the almost complete absence of the ubiquitous LD12 lineage (Alphaproteobacteria, Supplementary Figure S2) in this lake, similar to findings from other high mountain lakes (Urbach et al., 2007; Sommaruga and Casamayor, 2009; Xing et al., 2009; Salcher et al., 2011; Peter and Sommaruga, 2016). On the other hand, Opitutae of the VadinHA64 lineage (Verrucomicrobia) and diverse Betaproteobacteria (lineage GKS98, *Polynucleobacter* sp., *Aquabacterium* sp., *Polaromonas* sp., and *Methylotenera* sp.) were present in higher read numbers in Lake Augstsee. Viridiplantae, and therein especially Chlorophyceae (Chlamydomonadales and Sphaeropleales) were overrepresented in the eukaryotic dataset (Supplementary Figures S1, S2).

### Pronounced Coupling Between Prokaryotes and Eukaryotes

The similar clustering of prokaryotic and eukaryotic communities in our study (**Figures 4**, **7**) might be caused either indirectly by reacting to the same environmental factors or directly by biotic interactions. A strong impact of grazing by protists on the diversity of prokaryotes has already been shown in numerous studies (Šimek et al., 1995, 2006; Boenigk and Arndt, 2002; Pernthaler, 2005). Thus, selective feeding can lead to taxonomic shifts in the composition of microbes and affect these in the long term. Moreover, the type of prey might also have a severe impact on the composition of protists, as different bacterial taxa support the growth of different predators (Šimek et al., 2013; Grujcic et al., 2018). On the other hand, there are mutualistic relationships known between bacteria and phytoplankton. The region surrounding the phytoplankton cell, called the “phycosphere” provides a beneficial habitat for bacteria (Bell and Mitchell, 1972; Blackburn et al., 1996). Motile, chemotactic prokaryotes can swim towards substrate plumes surrounding the algal cell, where they profit from exudates and play an important role in the remineralization of nitrogen and phosphate (Stocker, 2012; Pernthaler, 2017). Non-motile bacteria may also benefit from phytoplankton blooms as released algal exudates diffuse in the water and represent an important carbon source (Salcher et al., 2015; Pernthaler, 2017; Neuenschwander et al., 2018). A temporal linkage of phytoplankton and prokaryotes has been described for various freshwater systems, where algal blooms are closely followed by maxima of distinct fast-growing bacteria affiliated with Bacteroidetes (e.g., *Flavobacterium* sp., *Fluviicola* sp.) or Betaproteobacteria (e.g., *Limnohabitans* sp. or other Comamonadaceae; Oxalobacteraceae) (Zeder et al., 2009; Eckert et al., 2012; Bižić-Ionescu et al., 2014; Šimek et al., 2014; Neuenschwander et al., 2015). However, the concentration of phytoplankton is not the only factor playing a major role; species composition is also of decisive importance, as some bacterial taxa show highly species-specific responses to the exudates of different algal taxa (Šimek et al., 2011; Paver et al., 2013; Horňák et al., 2017). Several studies have reported concordant temporal dynamics between primary producers and bacteria over a short period of the year (Kent et al., 2007; Paver et al., 2015; Woodhouse et al., 2016).

Our analyses over a broader time scale revealed similar patterns as the differences between prokaryotic and eukaryotic samples were relatively small (**Figure 8**). Patterns based on biotic interactions might be expected to be time-lagged and thus to deviate from patterns formed by responses to abiotic factors only. However, differentiating such time-lagged responses from direct responses would require a denser sampling as applied in our study. Even high-frequency samplings may not unequivocally allow to differentiate between the two scenarios as the rapidity of responses to abiotic factors may also systematically differ between prokaryotes and eukaryotes (Šimek et al., 2014). Even though abiotic reasons cannot be decoupled from biotic interactions we consider it likely that the observed patterns indicate that they probably follow the same environmental factors since the same environmental data corresponded to the clustering of both organismic groups (**Figures 3**, **6**). It might also be possible that other factors not measured in our study lead to the observed results or that they are indeed based on a strong linkage between prokaryotic and eukaryotic communities.

### Seasonal Variations of Microbes

Our time series analyses showed a linear correlation between time elapsed and increase in dissimilarity (**Figure 9**), in agreement with reports from other lakes (Eiler et al., 2012; Li et al., 2015). The correlation and its degree, however, differed for the analyzed lakes. Whereas Lake Augstsee showed a strong connection for both protists and bacteria, the correlation for bacteria in Lake Wallersee was not significant, and was also less pronounced for protists. The results for Lake Fuschlsee showed a recurring pattern over the 2 years of study. This proves that even if the community has a slightly different structure, they react similarly to the time factor. However, bacteria showed generally weaker correlations and smaller changes in dissimilarity over time (but this was not statistic significant), as indicated by lower slopes of the linear regression. Similar results were obtained in a study about subtropical reservoirs (Liu et al., 2015). This has been explained by high dispersal probability, high abundance, potentially high growth rates and rapid evolutionary adaptations (Liu et al., 2015). Also, long-term studies have shown that interannual variation is often greater than the level of seasonal changes for bacterioplankton communities (Tammert et al., 2015; Linz et al., 2017).

We repeatedly sampled and analyzed Lake Fuschlsee in the following year in order to test for recurrent patterns over the course of different seasons and to study the microbial OTU assemblage consistency within a lake. The fractions of shared OTUs between subsequent years were slightly higher as the fraction of shared OTUs between two separate lakes. This strong variation in OTU composition may be due to a strong random inoculation of the microbial plankton community from the seed bank stored in the sediment. Irrespective of the shifts in OTU composition, Lake Fuschlsee showed the same diversity patterns in both years with surprisingly high consistency, even though the autumn of the second year was not fully sampled. Long-term studies on lakes have shown a high consistency in phytoplankton communities, even over several decades (Berman et al., 1992; Anneville et al., 2002; Zohary, 2004; Salmaso and Padisak, 2007). However, changes in nutrient content or mixing regimes can have a major impact on sensitive species, and thus greatly change the whole community and the stability of the system (Salmaso, 2010; Posch et al., 2012; Yankova et al., 2017).

## Conclusion

Our study shows a pronounced coupling between bacteria and eukaryotes in seasonal samplings of three Austrian lakes. However, our temporal resolution (biweekly sampling) was not high enough to determine if this was caused by direct biotic interactions or by reacting to the same seasonally changing environmental forces. Future studies with higher temporal resolution need to sort out the share of independent similar responses of bacterial and eukaryotic communities to abiotic factors and of biotic interactions between the two groups possibly triggering a commutated community response.

## Data Availability Statement

The eukaryotic dataset analyzed for this study can be found in the NCBI database under the Bioproject Accession number PRJNA384347 (https://www.ncbi.nlm.nih.gov); the prokaryotic dataset under the Bioproject Accession number PRJNA445789.

## Author Contributions

CB and MS wrote the manuscript with input from all authors. MJ and RP conducted the bioinformatic analyses. CB, MJ, and MS performed the analysis. MJ verified the analytical methods. JB initiated and coordinated the study. All authors provided critical feedback and helped shape the research, analysis, and manuscript.

## Conflict of Interest Statement

The authors declare that the research was conducted in the absence of any commercial or financial relationships that could be construed as a potential conflict of interest.

## Footnotes

1 https://www.ncbi.nlm.nih.gov
