# Supplementary material for: Synchrony of Eukaryotic and Prokaryotic Planktonic Communities in Three Seasonally Sampled Austrian Lakes
Source: Front Microbiol. 2018 Jun 15;9:1290. doi: 10.3389/fmicb.2018.01290 (PMC6014231; doi:10.3389/fmicb.2018.01290)
Supplement: Supplementary file 1 [file Table_1.PDF]

Table S1. Details on sample dates and environmental conditions

Lake	sample code	sampling date	Temp [°C]	Cond [µS cm-1]	pH
Fuschlsee	FU 16	18.4.06	6.40	372.00	7.41
	FU 18	2.5.06	9.60	330.00	7.92
	FU 20	15.5.06	12.10	321.00	7.80
	FU 22	31.5.06	10.90	311.67	7.97
	FU 24	12.6.06	17.00	316.67	8.35
	FU 26	26.6.06	24.20	306.00	8.16
	FU 28	10.7.06	22.70	307.33	8.05
	FU 29	21.7.06	24.90	310.00	8.07
	FU 32	7.8.06	18.60	288.00	8.15
	FU 34	21.8.06	18.20	289.67	8.27
	FU 36	4.9.06	16.90	302.00	8.16
	FU 38	18.9.06	17.90	280.67	8.16
	FU 40	2.10.06	17.40	248.00	8.19
	FU 42	16.10.06	14.17	252.00	8.17
	FU 44	30.10.06	13.10	247.33	8.05
	FU 46	15.11.06	8.60	315.00	7.96
	FU 48	27.11.06	8.30	299.67	7.99
	FU 50	11.12.06	6.40	275.00	7.94
	Fu 13/07	28.3.07	5.20	323.00	8.05
	Fu16/07	16.4.07	11.0	263.33	8.23
	Fu19/07	7.5.07	14.4	312.67	8.4
	Fu22/07	30.5.07	15.2	309.00	8.17
	Fu25/07	21.6.07	21.5	317.33	8.15
	Fu28/07	9.7.07	18.4	292.00	8.34
	Fu31/07	31.7.07	19.6	288.67	8.2
	Fu34/07	22.8.07	20.0	256.33	8.15
	Fu37/07	13.8.07	15.5	267.67	7.5
Wallersee	WA 16	18.4.06	12.90	378.00	7.38
	WA 18	2.5.06	15.40	349.00	7.94
	WA 20	15.5.06	15.60	359.67	7.78
	WA 22	31.5.06	11.40	347.67	8.03
	WA 24	12.6.06	18.20	354.67	8.06
	WA 26	26.6.06	26.30	337.33	8.16
	WA 28	10.7.06	26.80	331.33	8.41
	WA 29	21.7.06	29.50	334.67	8.13
	WA 32	7.8.06	18.00	323.33	7.95
	WA 34	21.8.06	20.00	322.67	8.34
	WA 36	4.9.06	18.20	324.33	7.99
	WA 38	18.9.06	17.90	311.00	8.02
	WA 40	2.10.06	20.00	274.33	8.06
	WA 42	16.10.06	14.80	291.33	7.91
	WA 46	15.11.06	8.80	334.33	7.70
	WA 48	27.11.06	10.00	332.67	7.76
	WA 50	11.12.06	8.30	304.00	7.95
Augstsee	AU 28	12.7.06	15.60	73.33	7.48
	AU 30	24.7.06	20.20	76.00	7.71
	AU 32	8.8.06	10.00	94.00	7.38
	AU 34	22.8.06	12.00	86.67	7.70
	AU 36	6.9.06	16.90	94.00	7.63
	AU 38	19.9.06	10.00	91.67	7.33
	AU 40	4.10.06	11.30	101.67	7.64
	AU 42	17.10.06	10.50	83.67	7.55
	AU 44	31.10.06	8.10	93.00	7.15
