# Supplementary material for: Synchrony of Eukaryotic and Prokaryotic Planktonic Communities in Three Seasonally Sampled Austrian Lakes
Source: Front Microbiol. 2018 Jun 15;9:1290. doi: 10.3389/fmicb.2018.01290 (PMC6014231; doi:10.3389/fmicb.2018.01290)
Supplement: Supplementary file 2 [file Table_2.PDF]

Table S2. Chemical parameters for the three lakes

	Fuschlsee	Wallersee	Augstsee
Sample code	FU 48	WA 48	AU 44
Sample date	27.11.06	27.11.06	31.10.06
Temp [°C]	8.30	10.00	8.10
Cond [µS cm-1]	299.67	332.67	93.00
pH	7.99	7.76	7.15
Alkalinity/Gran [mequiv m-3]	2140.00	3340.00	1035.00
NO3-N [mg m-3]	456.00	798.00	77.00
SO4 [mg/l]	1.87	7.83	1.38
Cl [mg/]	0.69	7.83	0.68
NH4-N [mg m-3]	3.00	4.00	48.00
Na [mg/l]	1.10	4.80	0.45
K [mg/l]	0.21	1.18	0.43
Mg [mg/l]	2.63	7.43	0.54
Ca [mg/l]	44.96	62.54	23.46
DRSi [µg/l]	403.00	1845.00	254.00
TP [µg/l]	6.80	14.40	13.20
DOC [µg/l]	7436.00	4432.00	2809.00
DN (µg/l)	539.00	967.00	236.00
Turbidity		2.31	6.01
DP [µg/l]	1.80	8.50	2.40
