# Supplementary material for: Synchrony of Eukaryotic and Prokaryotic Planktonic Communities in Three Seasonally Sampled Austrian Lakes
Source: Front Microbiol. 2018 Jun 15;9:1290. doi: 10.3389/fmicb.2018.01290 (PMC6014231; doi:10.3389/fmicb.2018.01290)
Supplement: Supplementary file 3 [file Image_1.PDF]

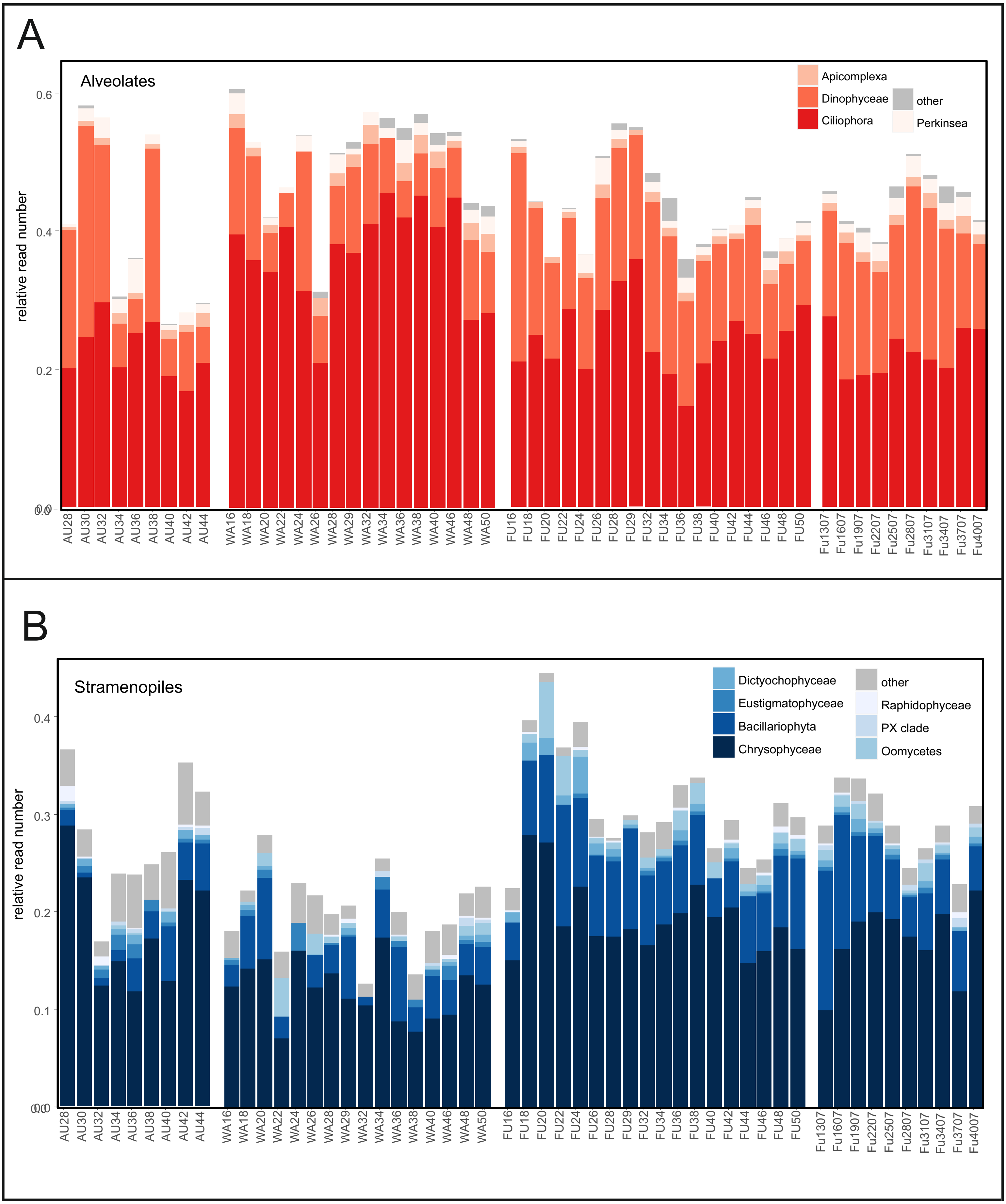

Figure S1. Details of main taxonomic groups of the analyzed eukaryotic samples based on Hellinger transformed rarefied reads. A: Alveolata; B:Stramenopiles; C:Viridiplantae; D: Chryptophyta.

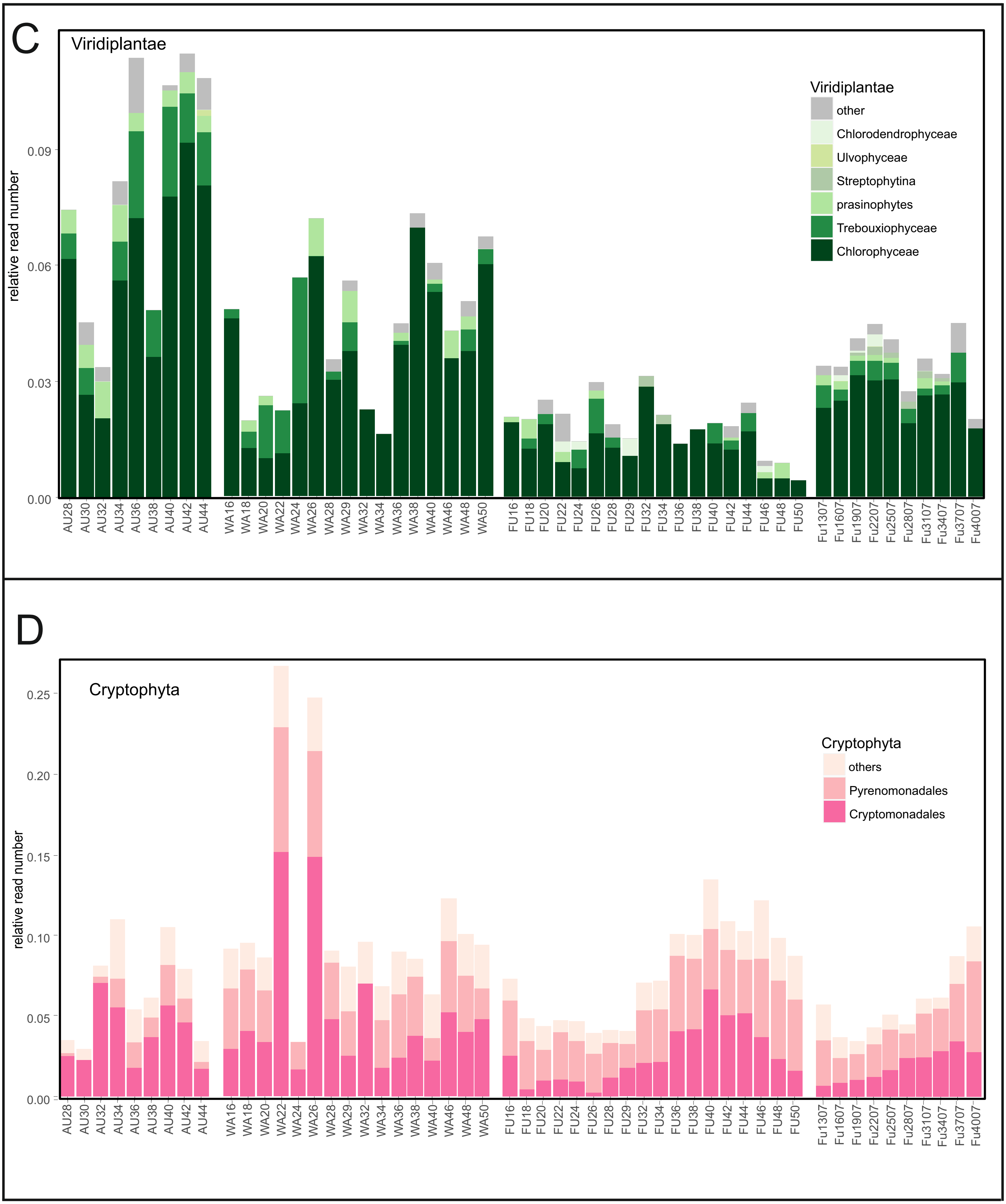

Figure S1. Details of main taxonomic groups of the analyzed eukaryotic samples based on Hellinger transformed rarefied reads. A: Alveolata; B:Stramenopiles; C:Viridiplantae; D: Chryptophyta.
