# Supplementary material for: Synchrony of Eukaryotic and Prokaryotic Planktonic Communities in Three Seasonally Sampled Austrian Lakes
Source: Front Microbiol. 2018 Jun 15;9:1290. doi: 10.3389/fmicb.2018.01290 (PMC6014231; doi:10.3389/fmicb.2018.01290)
Supplement: Supplementary file 4 [file Image_2.PDF]

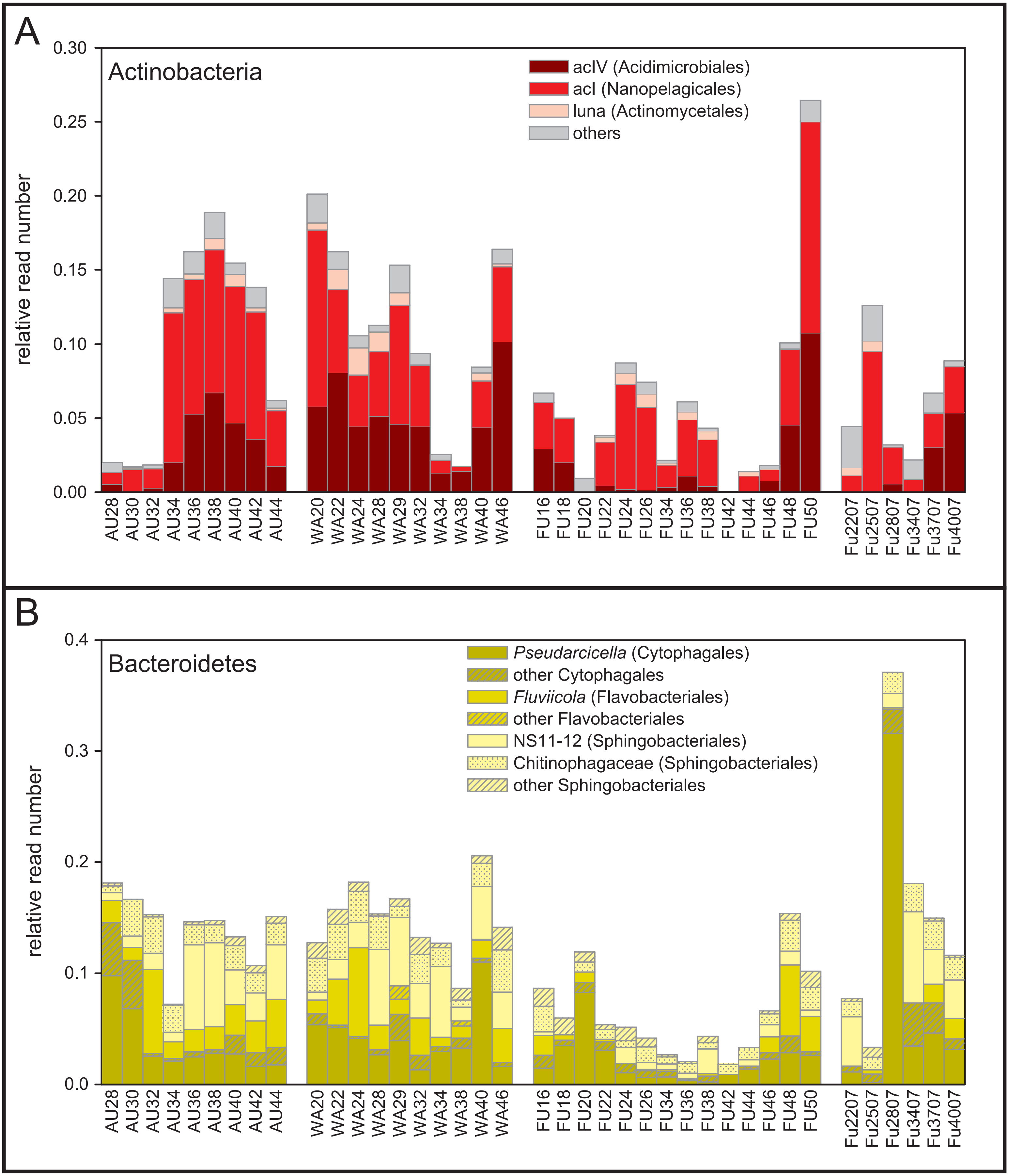

Figure S2. Details of main taxonomic groups of the analyzed prokaryotic samples based on Hellinger transformed rarefied reads. A: Alphaproteobacteria; B:Betaproteobacteria; C: Actinobacteria; D: Spingobacteria, E: Cyanobacteria; F: Verrucomicrobia.

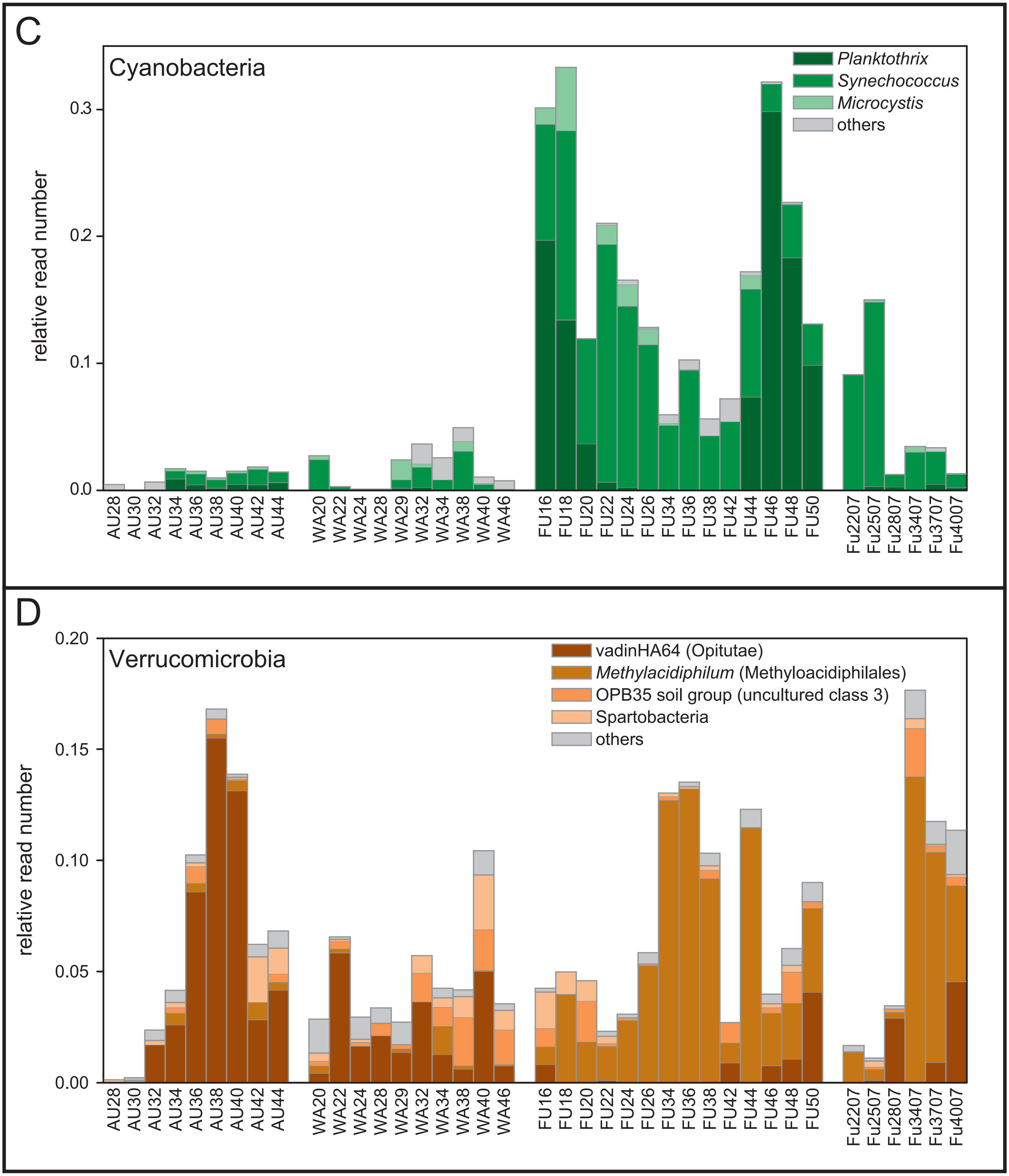

Figure S2. Details of main taxonomic groups of the analyzed prokaryotic samples based on Hellinger transformed rarefied reads. A: Alphaproteobacteria; B:Betaproteobacteria; C: Actinobacteria; D: Spingobacteria, E: Cyanobacteria; F: Verrucomicrobia.

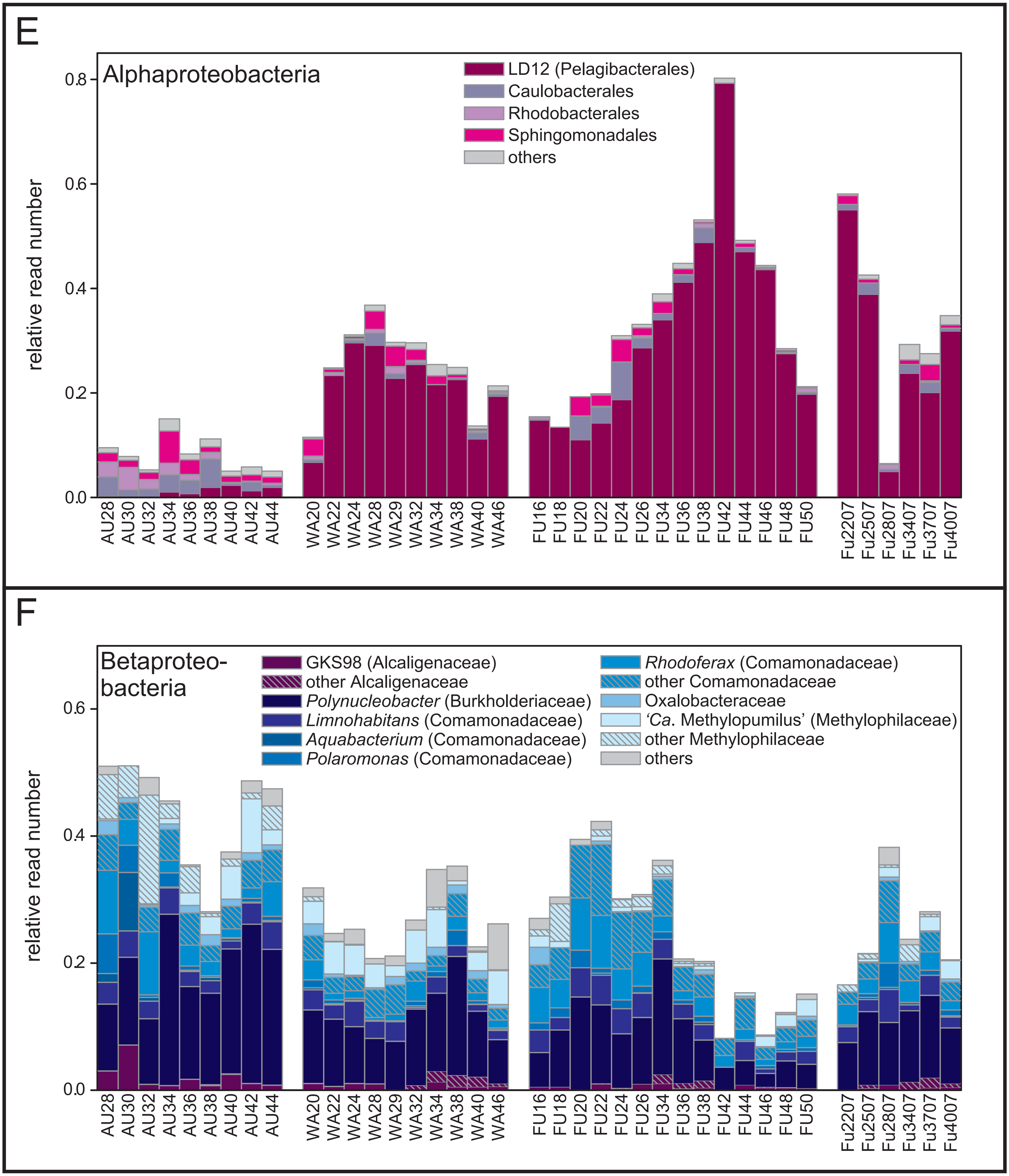

Figure S2. Details of main taxonomic groups of the analyzed prokaryotic samples based on Hellinger transformed rarefied reads. A: Alphaproteobacteria; B:Betaproteobacteria; C: Actinobacteria; D: Spingobacteria, E: Cyanobacteria; F: Verrucomicrobia.
