# Supplementary material for: Synchrony of Eukaryotic and Prokaryotic Planktonic Communities in Three Seasonally Sampled Austrian Lakes
Source: Front Microbiol. 2018 Jun 15;9:1290. doi: 10.3389/fmicb.2018.01290 (PMC6014231; doi:10.3389/fmicb.2018.01290)
Supplement: Supplementary file 5 [file Image_3.PDF]

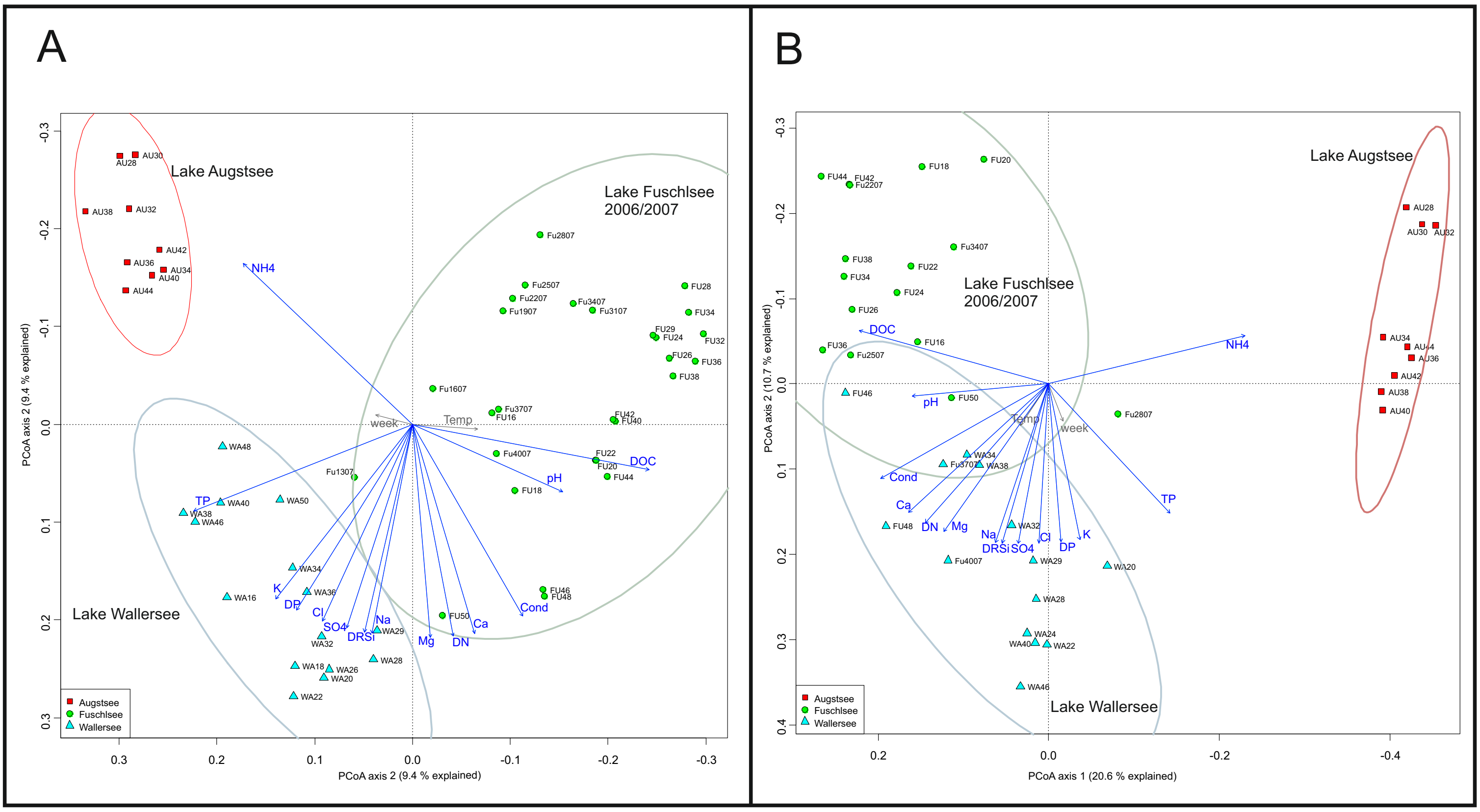

Figure S3. Community structure of the eukaryotic dataset by principle component analyses (PCoA). Environmental factors including only annual measured data applied a posterior. A:Eukarytic samples; B: Prokaryotic samples.
