# Supplementary material for: Synchrony of Eukaryotic and Prokaryotic Planktonic Communities in Three Seasonally Sampled Austrian Lakes
Source: Front Microbiol. 2018 Jun 15;9:1290. doi: 10.3389/fmicb.2018.01290 (PMC6014231; doi:10.3389/fmicb.2018.01290)
Supplement: Supplementary file 6 [file Image_4.PDF]

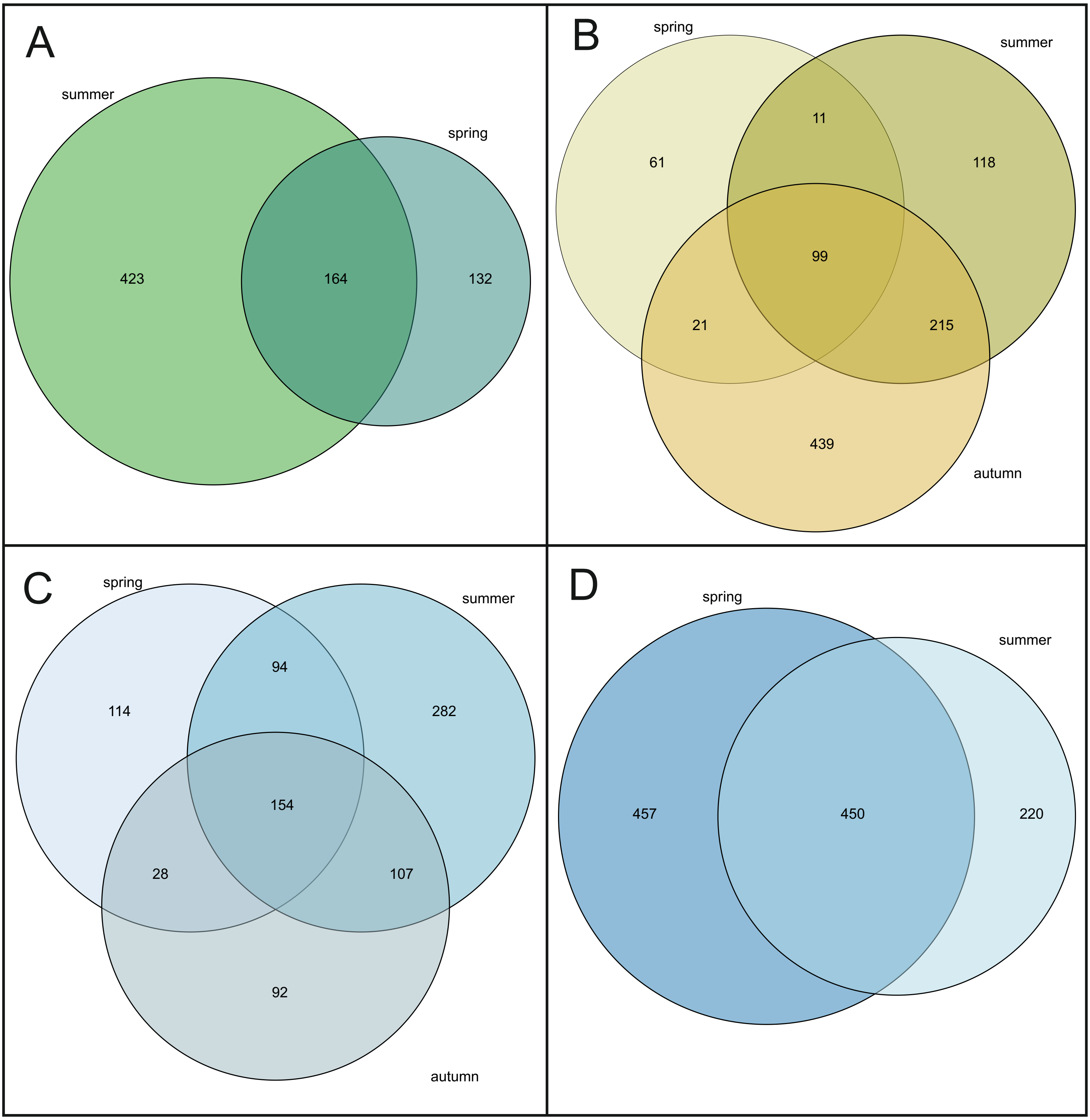

Figure S4. Diagram showing the overlap of occurring eukaryotic OTUs within the analyzed lakes. Compared are seasonal clusters based on the hierarchical clustering of Bray-Curtis dissimilarities
A: Lake Augstsee; B:Lake Wallersee; C:Lake Fuschlsee 2006; D: Lake Fuschlsee 2007.
