# Supplementary figures and images for: Synchrony of Eukaryotic and Prokaryotic Planktonic Communities in Three Seasonally Sampled Austrian Lakes
Source: Front Microbiol. 2018 Jun 15;9:1290. doi: 10.3389/fmicb.2018.01290 (PMC6014231; doi:10.3389/fmicb.2018.01290)

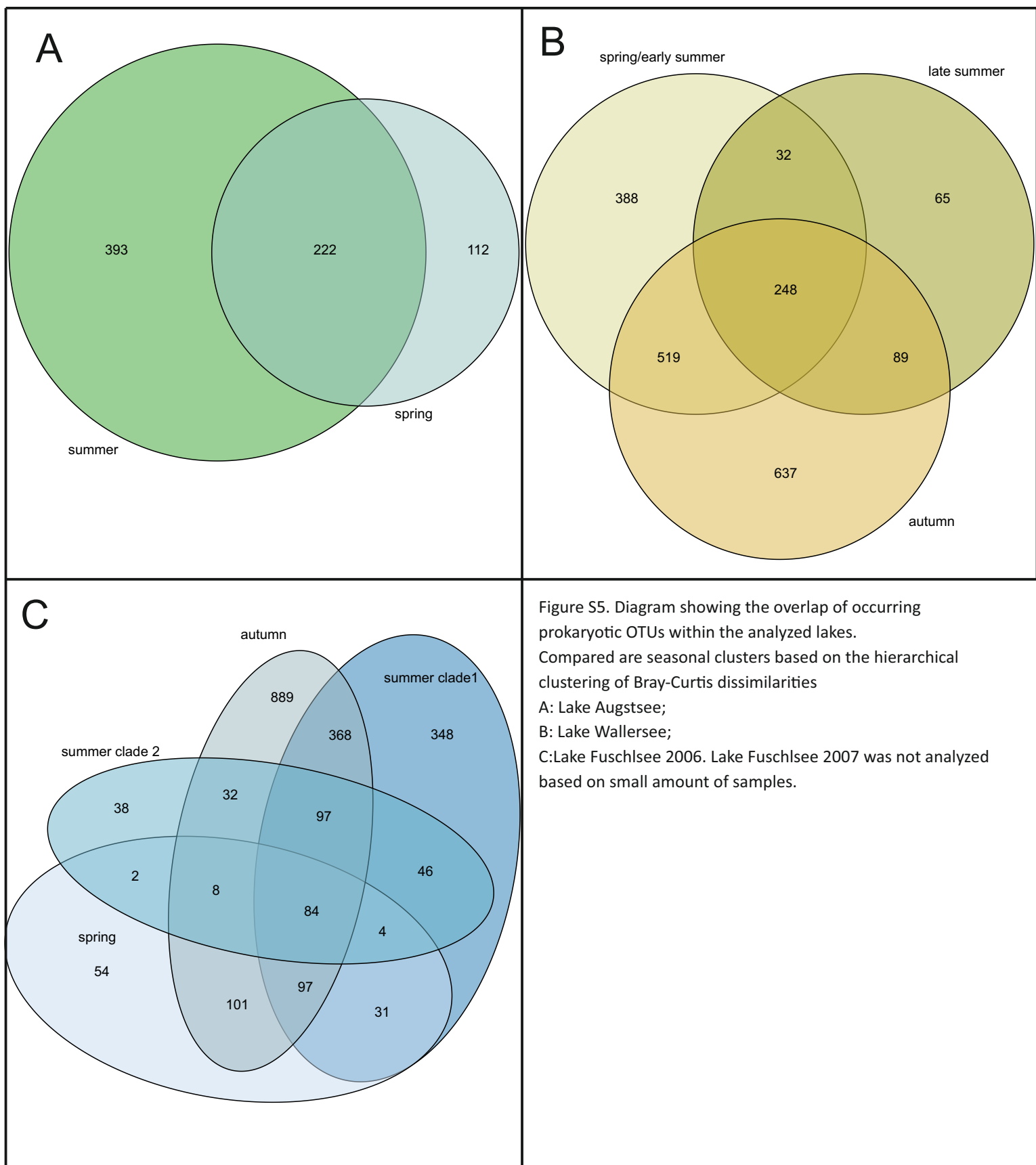

Supplement: Supplementary file 7 [file Image_5.PDF]
